# Green-Engineered Barrier Creams with Montmorillonite-Chlorophyll Clays as Adsorbents for Benzene, Toluene, and Xylene

**DOI:** 10.3390/separations10040237

**Published:** 2023-04-04

**Authors:** Meichen Wang, Timothy D. Phillips

**Affiliations:** Department of Veterinary Physiology and Pharmacology, College of Veterinary Medicine and Biomedical Sciences, Texas A&M University, College Station, TX 77843, USA

**Keywords:** barrier emulsion cream, adsorption isotherm, kinetics, BTX, ecotoxicological model, generally recognized as safe, chlorophyll, bentonite clay, topical application, dermal contact

## Abstract

Dermal exposures to hazardous environmental chemicals in water can significantly affect the morphology and integrity of skin structure, leading to enhanced and deeper penetration. Organic solvents, such as benzene, toluene, and xylene (BTX), have been detected in humans following skin exposure. In this study, novel barrier cream formulations (EVB^™^) engineered with either montmorillonite (CM and SM) or chlorophyll-amended montmorillonite (CMCH and SMCH) clays were tested for their binding efficacy for BTX mixtures in water. The physicochemical properties of all sorbents and barrier creams were characterized and were shown to be suitable for topical application. In vitro adsorption results indicated that EVB-SMCH was the most effective and favorable barrier for BTX, as supported by the high binding percentage (29–59% at 0.05 g and 0.1 g), stable binding at equilibrium, low desorption rates, and high binding affinity. Pseudo-second-order and the Freundlich models best fit the adsorption kinetics and isotherms, and the adsorption was an exothermic reaction. Ecotoxicological models using *L. minor* and *H. vulgaris* that were submersed in aqueous culture media showed that the inclusion of 0.05% and 0.2% EVB-SMCH reduced BTX concentration. This result was further supported by the significant and dose-dependent increase in multiple growth endpoints, including plant frond number, surface area, chlorophyll content, growth rate, inhibition rate, and hydra morphology. The in vitro adsorption results and in vivo plant and animal models indicated that green-engineered EVB-SMCH can be used as an effective barrier to bind BTX mixtures and interrupt their diffusion and dermal contact.

## 1. Introduction

The BTX contaminants consisting of benzene, toluene, and xylene are representative of hazardous, organic compounds that can be found in wastewater. These substances can cause serious health problems, such as irritation of the eyes, skin, and mucous membranes, leading to a weakened nervous system, decreased bone marrow function, and cancer (e.g., leukemia). The major sources of water contamination by BTX are wastewater discharge from industrial processes and chemical industries, release of petroleum products from the storage tanks, accidents associated with oil and gas spill activities, petroleum transportation, road pavement operations, and solvent use [1–3]. BTX are often found in municipal wastewater in the range of 0 to 933 g/L (85.5 g/L for toluene) [4], as well as during fermentation processes (around 3–4 g/L). In the acidophilic phase of the fermentation, toluene could increase up to 42,000 g/L [1]. Near industrial sites, a wide range of benzene has been detected in water samples from local monitoring wells (14 mg/L [5], 3–27 mg/L [6], and as high as 734 mg/L [7]).

The high motility of such hydrocarbons in soil-water systems is related to their relatively low octanol–water partition coefficient, which leads to slow soil absorption and preferential water transport, thereby resulting in the contamination of reservoirs [8]. Therefore, the problem with BTX in water can be magnified and complicated during disasters such as hurricanes and flooding, where BTX can be further mobilized and redistributed in the environment at contaminated sites, and be more readily available for skin penetration [9].

Extended dermal exposure to water can cause stratum corneum swelling and a more porous and permeable skin barrier [10–12]. Furthermore, exposure to environmental chemicals can weaken and damage the skin’s natural barrier, resulting in an increased amount and deeper penetration of chemicals, which, in turn, can affect the morphology and integrity of skin structure [12]. Specifically, the physical property of BTX allows for ready absorption across the skin, especially in compromised skin [13]. Moreover, these solvents may also enhance the penetration of other chemicals by disrupting the protective lipid layer of the skin [14]. Dermal exposure to BTX has been detected in farm workers and attendants exposed to solvents and heavy fuel oil [15–17]. Most efforts have been focused on developing effective remediation techniques for BTX in soil and groundwater, such as chemical degradation/oxidation processes, adsorption strategies using carbon, and various types of bioremediation. However, effective approaches to decrease skin contact to BTX in water have been limited to personal protective equipment (PPE) [18,19].

Barrier creams, such as sunscreens, can act as a shield to stop transepidermal water loss and block harmful contamination when applied onto the skin [20,21]. However, oxybenzone is one of the most widely used organic UVA filters in commercial sunscreen products due to its broad-spectrum UV coverage [22,23]. Despite established photoprotective effects, a number of important health issues have arisen, such as photoallergies and endocrine disruption [24–26]. Systemic absorption of oxybenzone has also been demonstrated, and the prevalence of exposure is estimated to be 97% in the general U.S. population [27]. Importantly, recent studies have detected benzene (which has been associated with leukemia) from oxybenzone in many commercial sunscreens [28,29]. Due to this public health concern, there has been an increased demand for the use of natural ingredients in sunscreens [22,30]. Since emulsions are the most common vehicles for barrier creams [30–33], a water-in-oil (W/O) emulsion containing only generally recognized-as-safe (GRAS) materials was developed in our laboratory as a base barrier formulation (Envirobloc^™^, EVB). EVB has shown properties that were suitable for topical application on the skin, such as high stability, spreadability, low rupture strength, and neutral pH. It has been shown to block the penetration of water to the skin and tightly adsorb water-soluble pesticides and per- and polyfluoroalkyl substances (PFAS) [34].

Additionally, clay-based sorbents have been included in creams for their protection against UV radiation ranging from 250–400 nm [35], increased adherence and water-resistance of skincare products, and more importantly, to interrupt the incursion of pollutants. For example, parent montmorillonite clay has been included in creams to treat diaper rash, dermatitis, and itchiness due to its adsorption of moisture, toxins, impurities, allergens, and bacteria [36–40]. Importantly, we have previously shown that chlorophyll-augmented montmorillonite clays significantly adsorb benzene through aromatic *π*–*π* stacking and alkyl–*π* interactions in water based on in silico computational simulations [41], and these clays have been included in the base EVB formulations for the binding of BTX and comparison to control montmorillonite clays in this study.

Dermal exposure to contaminants in water can cause serious issues for vulnerable populations, as well as medical personnel and first responders who are in contact with these pollutants for an extended period of time. Therefore, safe and effective strategies to prevent, or significantly reduce, skin contact with environmental chemicals from polluted flood waters are limited and critically needed. EVB creams will be economically feasible and can be used by underserved communities and first responders at the site of a disaster or emergency to enhance the efficacy of PPE in preventing dermal penetration of hazardous chemicals.

In this study, EVB formulations containing GRAS materials and clay-based sorbents were tested for the sorption of BTX mixtures in water using in vitro adsorption analyses and ecotoxicological models, including a BTX-sensitive plant (*L. minor*) and animal (*H. vulgaris*). We hypothesize that the topical application of EVB formulations that have been interfused with chlorophyll-amended clay can effectively bind BTX and interrupt skin uptake of BTX from contaminated water.

## 2. Materials and Methods

### 2.1. Preparation of Barrier Formulations

The W/O emulsion barrier formulation has been previously reported [34]. It contains hydrophobic components, such as zinc oxide, coconut oil, olive oil, shea butter and beeswax, that were emulsified with components such as glycerin and urea. Vitamin E and essential oils were added after the emulsion was mixed and cooled down to room temperature. These components are GRAS materials that are commonly used in other skincare products. Additionally, the base EVB cream was engineered with the inclusion of hydrated adsorbents at 5% (*w/w*) and mixed well to maintain a homogeneous texture [34]. These adsorbents were sieved through a 150 μm mesh. They included calcium montmorillonites (CM) from Engelhard Corp. (Cleveland, OH, USA), sodium montmorillonites (SM) from Source Clay Mineral Repository (Columbia, MI, USA) [42–44], and chlorophyll-engineered montmorillonites (CMCH and SMCH) that were synthesized as previously described [41].

### 2.2. Characterization of Sorbents

The density of sorbents was calculated by the difference between pycnometer weight divided by the sample volume (2 mL) [45]. The surface hydrophobicity was measured by the mass ratio of n-heptane and water vapor that were absorbed onto sorbents after 24 h at ambient conditions [46]. The coefficient of linear expansibility in water (COLE) was assessed by the difference in volume of sorbents following thorough equilibrium hydration and swelling for 24 h in water [47]. A higher ratio indicates greater hydration and expansion of the sample. The zeta potential and particle (hydrodynamic) size of 1 mg/mL clay suspension were measured three times by Zetasizer Nano ZC (Malvern, UK) at 25 °C. The pH was measured by a Pinnacle series M530P pH meter (Corning Electrochemistry, NY, USA). The photostability, purity, and chemical analysis of sorbents using X-ray fractionation (XRF) spectroscopy have been previously reported [41,44].

The fracture surfaces of sorbents were sputter-coated with 5 nm of Pt to image with a field emission scanning electron microscope (SEM) (JSM-7500F, JEOL, Peabody, MA, USA) at an operating voltage of 5 kV. The crystal structures of the sorbents were examined by XRD utilizing a Bruker D8 Endeavor with Cu-K α radiation (λ = 1.5406 Å) operating at 40 kV and 25 mA. Data collection was automated with a COMMANDER program by employing a DQL file, and analyzed by the program EVA. Fourier transform-infrared (FT-IR) spectra were obtained on an IR Prestige 21 system with a diamond attenuated total reflection (ATR) lens (Shimadzu Corp., Kyoto, Japan), and analyzed by the IRsolution v.1.40 software.

### 2.3. Characterization of Barrier Formulations

The barrier formulations were inspected visually for their color, homogeneity, texture, consistency, and phase separation. Homogeneity and texture were examined by rubbing a small quantity of the cream formulation for inconsistency and the presence of coarse undissolved materials [48]. The pH was determined by the Pinnacle series M530P pH meter (Corning Electrochemistry, NY, USA) on 1 g formulations in 100 mL of water at room temperature, and measured in triplicate. The physical stability of the barrier formulations was measured by subjecting 5 g of EVB, EVB-CMCH, and EVB-SMCH to three centrifugal cycles at 4000 rpm for 10 min in each cycle and observed for layer separation. The uniformity of weight was measured by filling and weighing 10 identical bottles with formulations [49,50]. To confirm the type of emulsion, the barrier formulations were diluted with oil and water and the diluents were observed for stability. The water in oil (W/O) type emulsion is stable on dilution with oil, but not stable with water [48]. To determine the shelf life of the formulated cream samples, they were kept intact in a closed and opaque container at room temperature in the dark for 6 months. The density, pH_pzc_, SPF, spreadability, and adhesiveness of the base EVB formulation have been previously reported [34].

### 2.4. Chemical Analysis

BTX concentrations were determined at room temperature on a Waters high-performance liquid chromatograph (HPLC) equipped with a 1525 binary mixing pump, a 717 plus autosampler, an XBridge C18 column (4.6 mm × 50 mm, 3.5 μm), and a 2487 UV/Visible detector (Waters^®®^ Corporation, Milford, MA, USA) [51]. An isocratic mobile phase of 66% acetonitrile and 34% water at 1 mL/min flow rate was programmed with an injection volume of 25 μL. Chemicals were qualified and quantified by the UV detector set at 254 nm and 202 nm. The retention time for BTX was between 1.2 min and 1.5 min. The Breeze^®®^ software was used to control the system and collect data. A linear standard curve of each chemical was derived using a blank mobile phase and 9 concentrations from 0.5 to 30 μg/mL in water (r^2^ = 0.999). The limit of detection and limit of quantification for BTX were 3.5 and 10.7 μg/mL, respectively.

### 2.5. Adsorption Screening

HPLC grade benzene, toluene, and o,m,*p*-xylene (purity ≥ 99%) were purchased from Fisher Scientific (Hampton, NH, USA). A chemical stock solution of BTX at 10 μg/mL for each chemical in pH 7 water was prepared weekly and sealed to reduce evaporation. This concentration of BTX was within the range of detected levels in polluted water to determine the maximum sorption efficacy of the EVB formulations. The EVB formulations were applied evenly on 15.8 mm filter papers (Millipore Corp., Badford, MA, USA) at doses of 0.05 g and 0.1 g and placed at the bottom of a 24-well plate. Chemical stock solutions at volumes of 2 mL were added to each well and mixed with EVB formulations on a rocking platform (VMR, Hamburg, Germany) at 600 rpm and 37 °C for 2 h, which corresponds to the suggested duration for reapplication of sunscreen products. Controls included 2 mL of blank (pH 7 water), chemical solution (10 μg/mL BTX), and EVB formulations in water, all of which contained a filter paper at the bottom of the well. All samples were filtered through Whatman filter papers (40 ashless, 11 cm) to discard EVB creams. The filtrate containing free chemicals was analyzed individually by HPLC, as mentioned above. The reduction rates of each chemical after treatment were calculated as the difference between chemical controls and treatment with EVB, EVB-CM, EVB-SM, EVB-CMCH, and EVB-SMCH at various doses.

### 2.6. Adsorption Kinetics

Adsorption kinetic models were included to study the effectiveness of EVB formulations in adsorbing BTX, following realistic exposure durations (within a few hours) [10]. To correlate the adsorption rate with concentrations and estimate the optimal dose of the adsorbent, 0.05 g EVB or EVB-SMCH on filter paper were placed at the bottom of the well and in contact with BTX mixtures in pH 7 water at various concentrations, i.e., 5, 8, and 10 μg/mL. These chemical/formulation mixtures in 24-well plates were sealed and agitated at 37 °C in pH 7 water for no longer than 4 h to limit BTX evaporation. The adsorption kinetics were analyzed by the adsorption of chemicals at different time intervals (10 min, 30 min, 1 h, 1.5 h, 2 h, and 4 h). To analyze the adsorption rate, pseudo-first-order, pseudo-second-order, and Elovich models [52,53] were investigated. The nonlinear pseudo-first-order rate equation is expressed as follows:

(1) $${\text{q}}_{\text{t}}{=\text{q}}_{\text{e}}\times \left[1-\text{exp}\left({-\text{K}}_{1}\times \text{t}\right)\right]$$

where *q*_e_ (mg/kg) and *q*_t_ (mg/kg) were the amounts of chemical adsorbed at equilibrium and at time t. K_1_ (min^−1^) was the rate constant of the first order. The pseudo-second-order kinetic model is expressed as:

(2) $${\text{q}}_{\text{t}}=\frac{\text{K}2\times {\text{qe}}^{2}\times \text{t}}{1+\text{qe}\times \text{K}2\times \text{t}}$$

where *q*_e_ and *q*_t_ were the amounts of the chemical adsorbed onto EVB formulations (mg/kg) at equilibrium and at time t (min), and K_2_ (mg/kg min) was the pseudo-second-order rate constant. The Elovich equation is one of the useful models for activated adsorption reactions [54], and is expressed as:

(3) $${\text{q}}_{\text{t}}=\frac{1}{\text{b}}\ln\;\text{ab}+\frac{1}{\text{b}}\;\ln\;\text{t}$$

where a was the initial adsorption rate (mg/kg min) and b was related to the extent of surface coverage and the activation energy involved in chemisorption (mg/kg). A trial-and-error procedure for computer operation for nonlinear models was used to determine the kinetic parameters by minimizing squared deviation and coefficient of determination between experimental data and predicted values [55].

Additionally, the effect of contact temperature on the kinetics was investigated by agitating the BTX mixture at 10 μg/mL per chemical and 0.05 g formulations at 3 temperatures, i.e., 4 °C, 24 °C, and 37 °C for 2 h.

### 2.7. Adsorption Isotherms

A BTX mixture at 10 μg/mL per chemical in pH 7 water was added to 25 mg EVB or EVB-SMCH formulations on 15.8 mm filter papers at the bottom of 24-well plates. Controls included 2 mL of blank solution, BTX in water, and EVB formulations in water, all in the presence of filter paper. The plates were sealed and agitated at 37 °C and 600 rpm using a rocking platform for 2 h. The formulation was then separated from the solution by filtering through filter papers (40 ashless, 11 cm). The filtrates containing free chemicals were detected using a Waters HPLC, as mentioned above.

The amount of bound chemical was determined by the concentration difference between control and test groups and expressed as mg/kg of EVB formulations. Table-Curve 2D (Systat Software, Inc., Palo Alto, CA, USA) and R programming that we previously developed and reported [56] were employed to plot adsorption data and calculate variable parameters. The R code was utilized to compute adsorption values and fitness to standard models based on maximum likelihood estimation, while standard deviations and confidence bands were calculated using the information matrix method [56,57]. The adsorption isotherms were generated by the Freundlich model based on mean values of independent triplicate. The Freundlich isotherm was used to describe the adsorption characteristics for a heterogeneous surface, as represented by the following equation:

(4) $${\text{q}}_{\text{e}}={{\text{K}}_{\text{f}}{\text{C}}_{\text{e}}}^{1/\text{n}}$$

K_f_ = Freundlich distribution constant, 1/n = degree of heterogenicity.

### 2.8. L. minor Assay

*Lemna minor* (duckweed) was procured from AquaHabit (Chatham, UK), and cultured under cool white fluorescent lights (400 ft-c intensity) with a light-to-dark cycle of 16 h/8 h at 25 °C. A mineral growth medium for *L. minor* was prepared based on the previous literature [58]. Three colonies of 3-frond lemna plants were randomly selected and incubated in sealed Pyrex dishes for 7 days. To assess toxicity, lemna was exposed to varying doses of BTX mixtures ranging from 10 to 0.5 μg/mL in 2 mL of culture media in each of the 24-well plates. For the detoxification study, a BTX solution at 10 μg/mL was treated with 0.2% EVBs (*w/v*) and agitated for 2 h before the filtrates were exposed to lemna for 7 days. Lemna was inspected daily for frond number and surface area of surviving plants, and analyzed by Image J (NIH, Bethesda, MD, USA). On Day 7, the plants were collected from individual dishes and homogenized in 1.5 mL of 80% acetonitrile. The chlorophyll content was extracted after 48 h (4 °C, dark) and quantified using UV/Visible scanning spectrophotometry (Shimadzu UV-1800, Kyoto, Japan) at 663 nm. Growth rate and inhibition % were calculated following standard OECD guidelines [58,59].


(5) $$\text{Growth rate}=\frac{\text{Log10}\;\left(\text{final frond}\right)-\text{Log10}\;\left(\text{initial frond}\right)}{\text{days}}$$

(6) $$\text{Inhibition of growth}\%=100\times \left(1-\frac{\text{frond in treatment}}{\text{frond in control}}\right)$$

### 2.9. H. vulgaris Assay

*Hydra vulgaris* were obtained from Environment Canada (Montreal, QC, Canada) and cultured at 18 °C., the morphology of the hydra was rated over time based on a hydra classification method [60] as an indicator of toxicity. The morphological scoring was classified using a dissecting microscope based on a 10-point scale, where a score of 10 represented normal, healthy hydra, and a score of 0 represented disintegrated (dead) hydra [60]. BTX mixtures at varying concentrations (4, 8 and 16 μg/mL) were exposed to hydra media to determine the minimum effective dose that resulted in 100% mortality of hydra in 5 days. Then, EVB formulations were included at 0.05% and 0.2% rates in the hydra media in a detoxification study. The chemical/sorbent complex in hydra media was mixed at 1000 rpm for 2 h before filtrates were exposed to hydra in sealed Pyrex dishes with parafilm. Three hydra colonies were included in each group and exposed to 4 mL of test media at 18 °C. The average score for each group was determined daily for 5 days. The final BTX concentrations were determined at the end of the 5-day experiment.

### 2.10. Statistical Analysis

All experiments included blanks and negative controls and were independently triplicated. Statistical significance was determined using a one-way ANOVA followed by a post hoc Tukey test. The reduction % in the screening study, mass of chemicals in the kinetic studies, and toxicity parameters in *H. vulgaris* and *L. minor* assays were calculated for standard deviation and *p*-value. Bonferroni correction was used for multiple test corrections [61]. Results were considered significant at *p* ≤ 0.05.

## 3. Results

### 3.1. Characterization of Sorbents

All montmorillonite sorbents were sieved through 150 μm mesh screens. Their bulk density and moisture percentage were within the range for bentonite clays (Table 1). The addition of lipophilic chlorophyll to the montmorillonite clays increased the surface hydrophobicity of the clay surface, enhancing the sorbent’s attraction for hydrophobic compounds [41]. The COLE (coefficient of linear expansibility) ratio indicates the expansibility of sorbents in water. The accuracy of this method was confirmed by the COLE values for CM and SM clays, which showed limited and high swelling, respectively. The addition of chlorophyll to SM showed reduced expansibility in water, possibly due to the cation exchange of Na^+^ that had low hydration energy. Additionally, the conjugation of light-sensitive chlorophyll with clay materials has been shown to increase their photostability [41].

**Figure 1.**
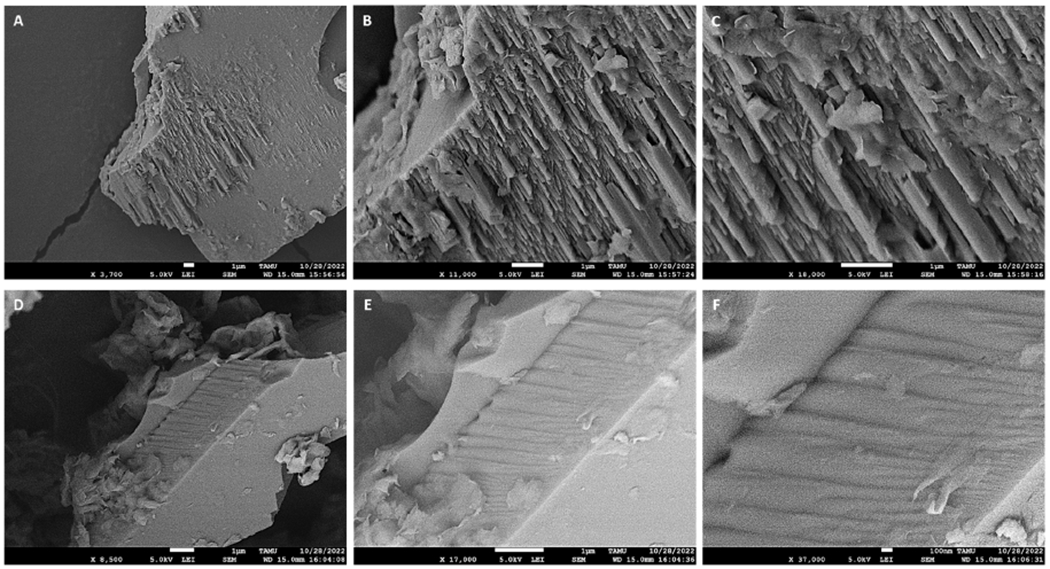
SEM images of CMCH (**A–C**) and SMCH (**D–F**).

The microscopic observations of CMCH and SMCH clays with zoomed-in views are shown in Figure 1. The SEM images showed aggregates of smooth surfaces with the typical widely layered structure of montmorillonite clays. Most of the aggregates showed broken edges and fractions, which might be due to grinding. Compared to parent montmorillonites [62], the amendment of chlorophyll showed no visual changes in structural morphology.

**Figure 2.**
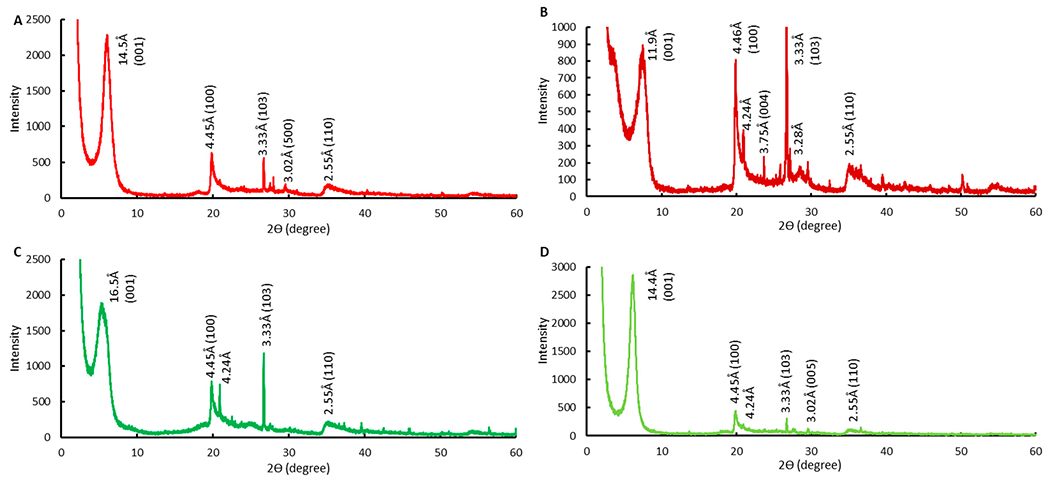
XRD patterns of CM (**A**), SM (**B**), CMCH (**C**), and SMCH (**D**).

The XRD patterns of montmorillonites, before and after modification with chlorophyll, are displayed in Figure 2. Montmorillonite was identified as the main compound by the characteristic peaks (2θ = 5.9°, 19.9°, 27.5°, 35.1°; or d = 14.5 Å, 4.5 Å, 3.2 Å, 2.6 Å). The presence of impurities was also shown by the slight peaks indicative of quartz (2θ = 20.9° and 26.7°) and calcite (2θ = 29.5°) [63,64], The d_001_ values of CM and SM were 14.5 Å and 11.9 Å, which can be attributed to the different interlayer cations (Ca^2+^ = 1.97 Å, Na^+^ = 0.95 Å), and the d_001_ was within the range of interlayer spacing for representative montmorillonite clays. Importantly, the basal spacing for CMCH increased following augmentation with chlorophyll to 16.5 Å, and SMCH was increased to 14.4 Å. These results indicated that chlorophyll acts to prop the layer-lattice structure resulting in a larger basal spacing between active interlayer surfaces. This work is supported by our previous molecular modeling work showing that chlorophyll forms aggregate in clay layers.

**Figure 3.**
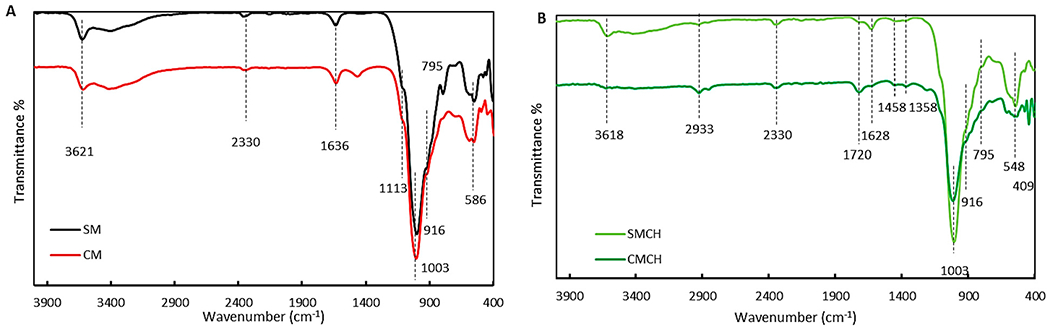
FT-IR spectra of parent CM and SM (**A**) and CMCH and SMCH (**B**).

The FT-IR spectra confirm and complement the SEM and XRD data. The two bands at 3621 cm^−1^ (Al–Al–OH stretching vibration) and 916 cm^−1^ (Al–Al–OH bending vibration) were typical for dioctahedral smectites [65] (Figure 3A). The band at 3620 cm^−1^ attributed to inter-octahedral Al/Mg–OH stretching, suggesting that the smectite was Al- and Mg-rich [65]. The 1636 cm^−1^ band corresponded to −OH bending vibration of water molecules [66], showing the hydrous nature of this clay material [67], The Si–O vibrations in-plane and out-of-plane were observed at 1003 cm^−1^ and 1113 cm^−1^, respectively. The weak peak at 795 cm^−1^ in the SM sample can be attributed to Si–O stretching of quartz and silica [68]. Si–H silane was observed at 2330 cm^−1^ [69]. The slight band around 2350 cm^−1^ was from room CO_2_ [70,71]. The amendment of chlorophyll to both CM and SM clays was confirmed by the occurrence of new bands at 2933 cm^−1^, and 409 cm^−1^ and 1720 cm^−1^, which were attributed to C-H vibration in chlorophyll, and the C–C=O bond of the carbonyl group in chlorophyll vibration outside the plane, respectively [71] (Figure 3B). The amended clays maintained the characteristic bands of montmorillonite clays.

### 3.2. Characterization of Barrier Formulations

All barrier formulations in this study exhibited suitable characteristics for topical application. Base EVB formulations were white, creamy, and shiny in appearance, while EVB-CMCH and EVB-SMCH showed a light green color. They were smooth, opaque, glossy, and greasy on application. The dilution test confirmed that they were water-in-oil emulsions as they were stable and could dilute with oil but not aqueous solvents. Creams were uniform in weight with a standard deviation in weight within 10%, and their density was around 1.12 ± 0.1 g/mL. The pHs of EVB and EVB-SMCH were 6.48 and 8.82, respectively, which were compatible with the skin. No layer separation after centrifugation and no coarse, undissolved materials were observed. Additionally, the formulations were readily applicable and sheared evenly onto skin, and no visible difference in color or texture was observed after washing the skin under running water for 10 min, suggesting high adhesiveness. According to the scientific literature, these are desirable features for cosmetic products intended for skin application [48–50].

### 3.3. Adsorption Screening

**Figure 4.**
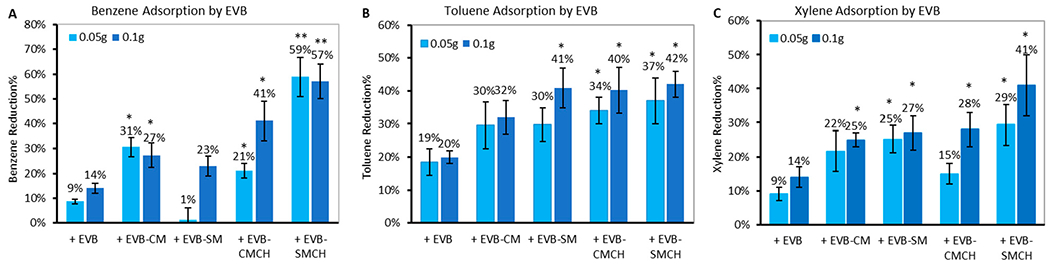
The percent reduction in the adsorption of (**A**) benzene, (**B**) toluene, and (**C**) xylene by various EVB formulations for 2 h (* *p* ≤ 0.05; ** *p* ≤ 0.01 compared to base EVB).

The binding results for BTX onto surfaces of base EVB and EVB containing clay-based sorbents are shown in Figure 4. The base EVB formulation showed limited adsorption of BTX, with reduction percentages of less than 20%; whereas the inclusion of previously hydrated CM and SM clays in EVB increased the attraction of BTX to active binding sites. Furthermore, the inclusion of 5% chlorophyll-amended clays (CMCH, and especially SMCH) significantly enhanced the binding efficacy, with the highest removal rates for BTX to 29–59%, compared to EVB alone and EVB-CM and EVB-SM. This is in alignment with our previous in vitro adsorption and in silico computational simulations, suggesting that chlorophyll-amended clays showed favorable binding profiles (52% binding) and thermodynamics for benzene through aromatic π–π stacking, alkyl–π interactions, and Van der Waals attractions [41]. Additionally, the inclusion of EVBs at 0.1 g showed similar or slightly higher chemical reduction than at the 0.05 g rate, showing stable and consistent binding. Based on the screening results, EVB-SMCH was the most effective formulation for BTX, and was included in the following adsorption studies in comparison to base EVB.

### 3.4. Effect of Chemical Concentration and Contact Time on Adsorption

**Figure 5.**
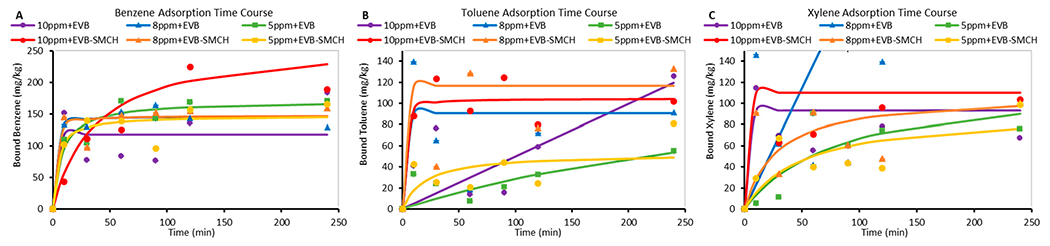
Effect of initial chemical concentrations and contact time (for up to 4 h) on the adsorption of (**A**) benzene, (**B**) toluene, and (**C**) xylene in 0.05 g EVB and EVB-SMCH at 37 °C and pH 7.

A time course study was carried out with BTX at varying concentrations and with the inclusion of EVB and EVB-SMCH at 37 °C and pH 7. The total reaction time was 4 h to ensure that reaction equilibrium was reached and to limit BTX evaporation. The results from the time course of adsorption are shown in Figure 5. The adsorption onto base EVB showed both linear (e.g., with 10 and 5 μg/mL toluene and 8 μg/mL xylene) and curved lines as plotted by pseudo-first- and second-order, respectively, suggesting that different sites and mechanisms may be involved on EVB surfaces. When EVB showed a curved binding plot, the maximum amounts of bound chemicals were less than with EVB-SMCH at equilibrium, especially at higher chemical concentrations (10 and 8 μg/mL BTX), whereas the adsorption plot for EVB-SMCH showed a consistent curved shape at all BTX concentrations, as plotted by pseudo-second-order. Importantly, BTX remained bound on EVB-SMCH and the adsorption capacity achieved a constant value after equilibrium, suggesting that the adsorption interactions onto EVB-SMCH were stable with limited dissociation. In most cases, the adsorption of BTX onto EVB-SMCH occurred quickly within 10 min, and reached equilibrium within 20 min, indicating high binding affinity.

### 3.5. Kinetic Models

Kinetics and equilibrium of the adsorption process were determined to evaluate adsorption dynamics and determine the rate determining step and mechanism of adsorption behavior. The above time course results were analyzed by three common nonlinear kinetic models, including pseudo-first-order, pseudo-second-order, and Elovich. Based on the adjusted correlation coefficient values (R^2^ ≥ 0.87) and the comparison between the binding capacity derived from experiments (*q*_e_, exp) and calculated from the models (*q*_e_, cal), the adsorption of BTX onto EVB-SMCH best fit the pseudo-second-order kinetic model for all chemicals at all concentrations. Therefore, the parameters for the pseudo-second-order kinetic model are shown in Table 2. This suggested that the adsorption kinetics onto EVB-SMCH were mainly dependent on diffusion-limited processes and affected by heterogeneous distributions of pore sizes and continual partitioning of BTX. The driving force for BTX adsorption to EVB-SMCH was the concentration gradient, and therefore, the rate of adsorption was proportional to the driving force or the square of the driving force [70]. The expected effect of green-engineered barrier formulations (EVB-CMCH and EVB-SMCH) will be to adsorb and block the dermal penetration of pollutants at the surface of the cream. Barrier creams after application for up to 4 h can be washed off by soaps, and the water can be collected for further remediation, whereas the adsorption of BTX onto base EVB fit both the pseudo-first-order and the pseudo-second-order models based on a wide range of R^2^ for pseudo-second-order (0.38 to 0.99) and no maximum binding capacity. This suggested that BTX binding onto EVB involved multiple mechanisms, and was subject to dissociation.

### 3.6. Effect of Temperature

**Figure 6.**
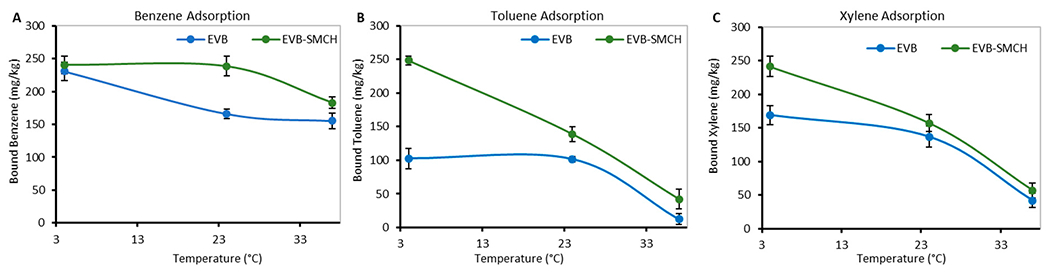
Effect of contact temperature on the adsorption of (**A**) benzene, (**B**) toluene, and (**C**) xylene on 0.05 g EVB and EVB-SM in pH 7 water for 2 h.

To study the impact of temperature on the kinetics of adsorption, the chemical/formulation complexes were reacted in pH 7 water for 2 h at three different temperatures, i.e., 4 °C, 24 °C, and 37 °C. As shown in Figure 6, the amount of BTX adsorbed onto EVB and EVB-SMCH decreased as the temperature increased. This trend suggested that the adsorption of EVB and EVB-SMCH was exothermic (release of heat). Another possible reason contributing to the lower binding at higher temperatures was the slight increase in chemical solubility in water, reducing the interaction with EVB formulations. Importantly, the amount of bound BTX onto EVB-SMCH was higher than that on EVB at all temperatures, which was consistent with the results for binding capacity from the time course study and binding percentage from the screening study.

### 3.7. Adsorption Isotherms

**Figure 7.**
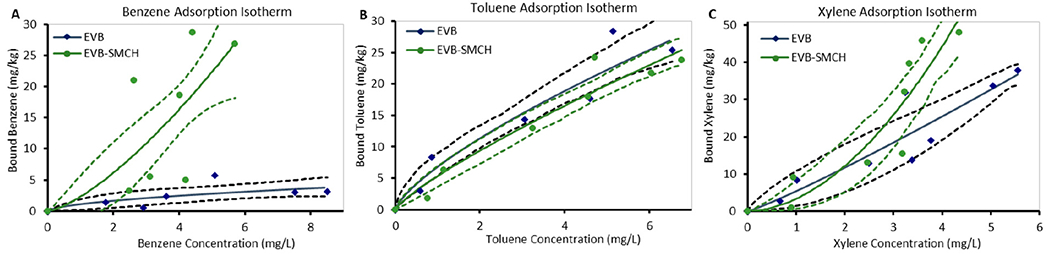
Adsorption isotherms of (**A**) benzene, (**B**) toluene, and (**C**) xylene on surfaces of 25 mg EVB and EVB-SMCH at 37 °C in pH 7 water for 2 h, plotted by the Freundlich model. Data represent the mean adsorption (mg/kg) at each concentration, run in triplicate. Bands indicate 95% confidence intervals on the mean response.

Isothermal data reflecting chemical adsorption onto EVB and EVB-SMCH surfaces were plotted in a Freundlich model, indicating heterogeneous binding sites (Figure 7). Based on the International Union of Pure and Applied Chemistry (IUPAC) classification of isotherms for the purpose of structural characterization, the adsorption isotherms of BTX were classified as either Type III or I, indicating surface binding interactions [72,73]. The values of Freundlich constants K_f_ and n were obtained from the plots of C_e_ versus *q*_e_, and are shown in Table 3. The larger value of n (smaller value of 1/n) implies a stronger and more favorable interaction between the adsorbent and adsorbate. Specifically, the reaction was favored when n > 0.68 (1/n < 1.46) [70], and adsorption of BTX onto EVB-SMCH all fit in this range. The numerical value of 1/n < 1 indicated that adsorption capacity was only slightly suppressed at lower equilibrium concentrations. This isotherm did not show any saturation of the adsorbent, thus infinite surface coverage was predicted mathematically, indicating multilayer adsorption on the surface [74]. The adsorption showed high reproducibility and high fitness to the Freundlich model, as supported by R^2^ ≥ 0.85 and 95% confidence interval bands from independent triplication.

### 3.8. L. minor Assay

*Lemna minor* is an aquatic plant that is sensitive to environmental chemicals. Its toxicological testing protocols are well-established, and have been widely used in ecotoxicology studies. Therefore, *L. minor* was used as a toxicity indicator in this study. In our preliminary study, filtered EVB formulations were included in the media at 0.2% to 1% to determine their relative safety. At 0.2% and 0.5% inclusions, EVB, EVB-CMCH and EVB-SMCH showed no significant difference in growth compared to the media control in terms of changes in frond number and surface area, chlorophyll content, and overall growth and inhibition rates (Figure S1), whereas base EVB inclusion at 1% delivered less growth, as shown by the 32%, 25%, and 11% decrease in frond number, surface area, and chlorophyll content compared to media, respectively. It also showed a significant decrease in growth rate versus the media control (*p* ≤ 0.05) (Figure S1). The results indicated the relative safety of the inclusion of EVB formulations at levels below 1%.

**Figure 8.**
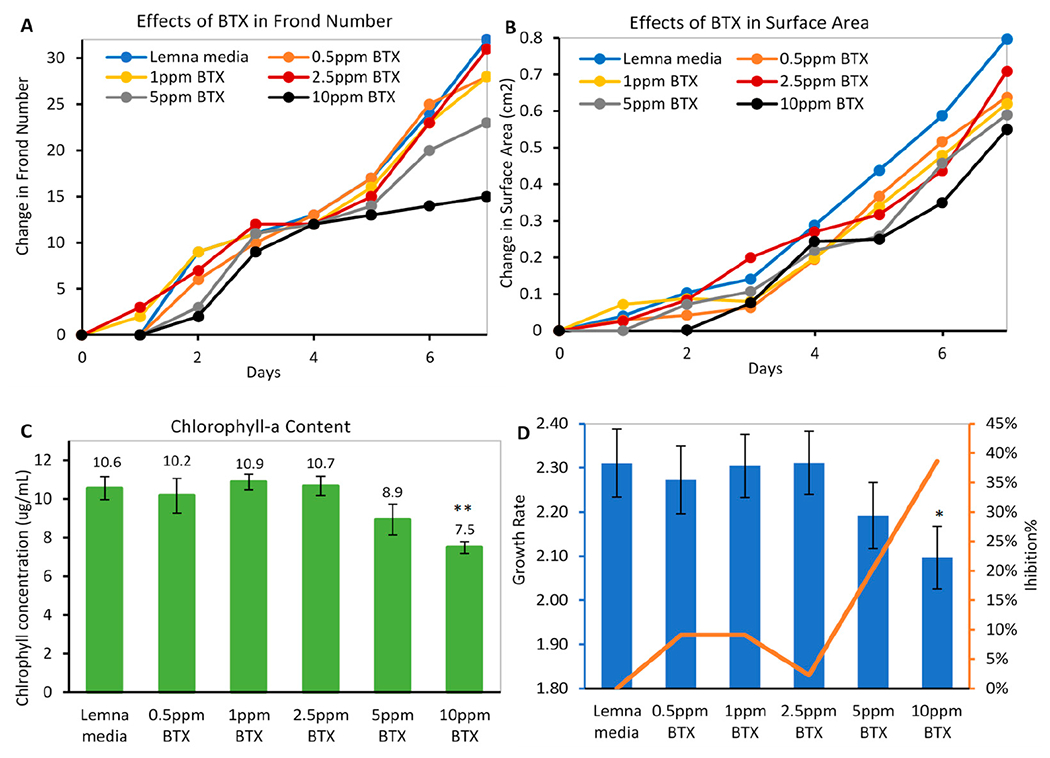
Dose-dependent toxicity of BTX on *L. minor* (**A**) frond number, (**B**) surface area of surviving plants, (**C**) chlorophyll content on Day 7, and (**D**) growth rate (bar graph) and inhibition percentage (line) (* *p* ≤ 0.05, ** *p* ≤ 0.01 compared to media control).

In a 7-day study, the lemna media supported growth by increasing 32 leaflets in frond number and increasing 0.8 cm^2^ in surface area. Plants exposed to BTX at various concentrations showed toxicity in a dose-responsive manner. Specifically, *L. minor* at high exposure concentrations (10 and 5 μg/mL BTX) only increased by 15 and 23 fronds (Figure 8A) and 0.55 and 0.59 cm^2^ in surface area (Figure 8B), respectively, whereas BTX at lower concentrations (0.5–2.5 μg/mL) slightly decreased growth compared to the media control. The chlorophyll-a content was extracted on Day 7 and detected using a UV–Vis scanning spectrophotometer. As shown in Figure 8C, the decreased chlorophyll-a content correlated with BTX concentrations, where 10 and 5 μg/mL BTX showed the least chlorophyll content. This aligned with visual observations, where highly exposed fronds were chlorotic with brown and white edges and altered morphologies, and produced fewer daughter fronds. The daily frond number was used to calculate an average growth rate and inhibition percentage. Similar to the above phenotypes, higher concentrations of BTX (e.g., 10 μg/mL) showed a lower growth rate (1.82) and a higher inhibition (38.6%) (Figure 8D) compared to media control, and lower BTX concentrations. All of the toxicity parameters in lemna were highly correlative, suggesting that lemna was a sensitive model to indicate BTX toxicity in water. Therefore, BTX at 10 μg/mL was used in the following treatment study.

**Figure 9.**
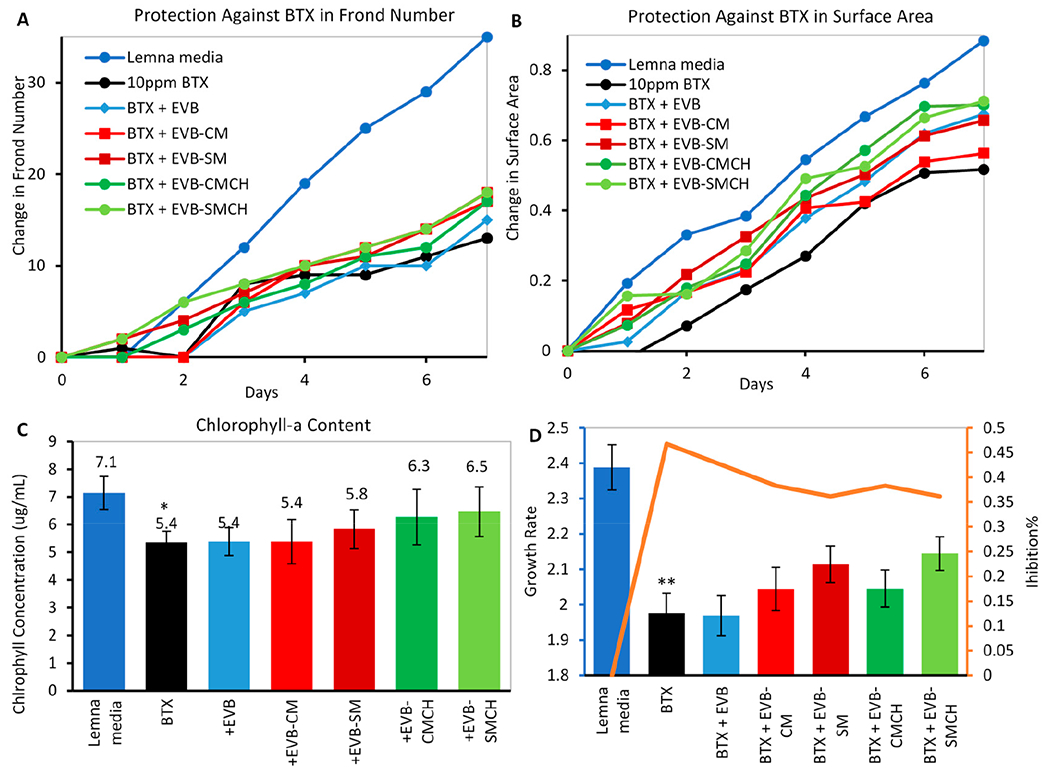
Protection against BTX by EVBs at 0.2% in the *L. minor* medium on (**A**) frond number, (**B**) surface area of surviving plants, (**C**) chlorophyll content on Day 7, and (**D**) growth rate (bar graph) and inhibition percentage (line) (* *p* ≤ 0.05, ** *p* ≤ 0.01 compared to media control).

All EVB formulations were individually included in BTX-exposed media at a 0.2% rate (*w/v*). The inclusion of all EVB formulations, including EVB, EVB-CM, EVB-SM, EVB-CMCH, and EVB-SMCH protected lemna growth by increasing the frond number and surface area, compared to BTX chemical controls (Figure 9A,B). Chlorophyll-a content, EVB-CMCH and EVB-SMCH showed the highest chlorophyll concentration at 6.3 and 6.5 μg/mL, respectively (Figure 9C). The average growth and inhibition rates calculated from the 7-day experiment showed that EVB-SMCH was the most effective barrier with the highest growth rate and lowest inhibition rate (Figure 9D). The results of all growth parameters collectively showed that the green-engineered EVB-SMCH at 0.2% inclusion adsorbed BTX more effectively than base EVB and other EVB formulations over the 7-day experiment, and most significantly, protected lemna from BTX toxicity.

### 3.9. H. vulgaris Assay

**Figure 10.**
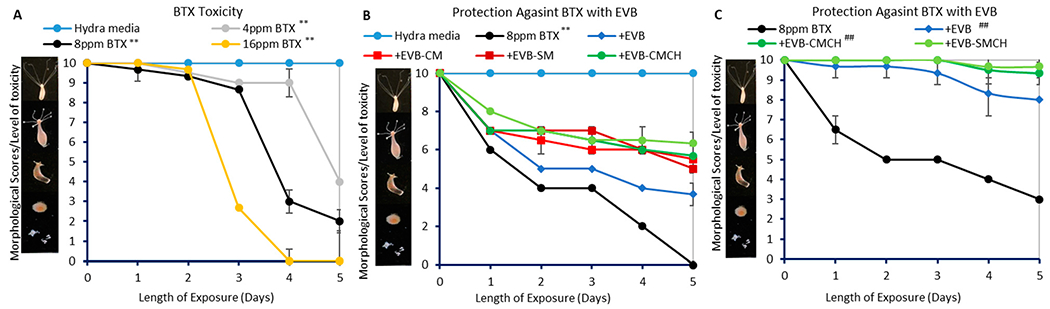
Hydra toxicity from BTX (**A**) and protection by EVBs at 0.05% (**B**) and 0.2% (**C**). The hydra media control showed consistent scores of 10. Data represent the mean morphological score at each time point, run in triplicate (** *p* ≤ 0.01 compared to media control; ^##^ *p* ≤ 0.01 compared to BTX).

*Hydra vulgaris* is very sensitive to environmental toxins, and has been widely used to indicate the toxicity of water pollutants. The inclusion of EVBs at 0.05–0.5% in the hydra media showed negligible difference (≤7%) in morphology changes compared to media control (score 10), suggesting that EVBs at less than 0.5% were safe for *H. vulgaris* after exposure (Figure S2). Therefore, *H. vulgaris* was included in this study to predict the protection of EVBs against BTX toxicity. As shown in Figure 10A, the morphology response of hydra to BTX at concentrations ranging between 4 to 16 μg/mL was dose-dependent, where 4 μg/mL BTX showed moderate toxicity, while 16 μg/mL showed rapid and complete mortality at the end of the experiment. Therefore, 8 μg/mL BTX was used in the sorbent treatment study to validate the efficacy and safety of sorbents. In Figure 10B, all EVB formulations were included at only 0.05% rates in the media, and showed significant protection (36.7–63.3%) against toxicity (*p* ≤ 0.01), with the green-engineered EVB-CMCH and EVB-SMCH showing the highest toxicity reduction (≥56.7%). At the end of the experiment, supernatants in the media were tested on HPLC for BTX concentrations. The final concentration in the BTX control averaged 5.8 μg/mL for each chemical, showing a 27.5% loss after 5 days due to their high volatility. The concentrations in the EVB-CMCH and EVB-SMCH averaged 3.2 and 2.1 μg/mL, respectively, corresponding to less toxicity, shown as the higher hydra morphology scores. Additionally, the inclusions of EVB, EVB-CMCH, and EVB-SMCH at a higher dose of 0.2% resulted in higher percentage protection, i.e., 71 ± 3%, 90 ± 6%, and 95 ± 6%, respectively (Figure 10C). This dose-dependent reduction in toxicity correlated with the Freundlich isothermal model showing sufficient but unsaturated binding sites on EVB surfaces.

## 4. Conclusions

Exposure to water can cause stratum corneum to swell and enhance skin permeability to hazardous chemical contaminants such as BTX. These pollutants can be further mobilized and redistributed in flood waters during disasters, thus threatening the health of people and animals onsite. Therefore, the development of barrier formulations with the potential to maintain the integrity of the skin and reduce dermal exposure to environmental chemicals is warranted. In this study, the adsorption efficacy and safety of a W/O emulsion formulation (EVB) with the inclusion of various clay-based sorbents were tested for BTX mixtures in water. The properties of active sorbents and sorbent formulations were characterized, and suggested the intercalation of chlorophyll into montmorillonite clays, and that EVB formulations were suitable for topical application onto the skin. Integrated in vitro adsorption screening, kinetics, and isothermal studies showed that the homogeneous inclusion of novel montmorillonite–chlorophyll clays within base EVB formulations significantly increased the adsorption of BTX mixtures. This was supported by high binding percentages, equilibrium within 20 min of contact, stable binding with no observable dissociation, and high affinity. The adsorption onto EVB-SMCH fit pseudo-second-order and Freundlich models, and the kinetic studies with varying temperatures predicted an exothermic reaction. In vivo ecotoxicological models using *L. minor* and *H. vulgaris* showed that the inclusion of EVB-SMCH in the culture media significantly reduced the BTX toxicity in a dose-dependent manner based on multiple growth endpoints, including plant frond number, surface area, chlorophyll content, growth rate, inhibition rate, and hydra morphology. Collectively, these EVB barrier formulations show promise for topical applications by first responders and vulnerable populations residing near contaminated sites to reduce common chemical exposures from flood waters following disasters. They may also be applied as “green-engineered” sunscreens, with value-added protection from environmental chemicals.

## Supplementary Material

Supplementary Material

## Figures and Tables

**Table 1.** Important physicochemical properties of montmorillonite sorbents.

	CM	SM	CMCH	SMCH
Appearance	Off-white to greyish-green powder	Off-white powder	Green powder	Green powder
pH	8.07	9.39	8.10	8.04
Zeta potential	−7.79 ± 0.70 mV	−22.3 ± 0.52 mV	−11.6 ± 2.22 mV	−14.2 ± 0.34 mV
Particle size	743.6 nm	670.7 nm	686.9 nm	775.6 nm
Bulk density	979.6 kg/m^3^	1109.6 kg/m^3^	758.4 kg/m^3^	1111.8 kg/m^3^
Moisture	10%	6%	6%	4%
Hydrophobicity	0.57	0.41	1.27	1.8
COLE	1.8	7.5	2	2.1

**Table 2.** Adsorption constants onto EVB and EVB-SMCH calculated by the nonlinear pseudo-second-order model and the experimental binding capacities for BTX at different concentrations.

Chemical	Cone. (C_i_, mg L^−1^)	EVB				EVB-SMCH			
		q_e_ (exp, mg kg^−1^)	q_e_ (cal, mg kg^−1^)	K_2_	R^2^_adj_	q_e_ (exp, mg kg^−1^)	q_e_ (cal, mg kg^−1^)	K_2_	R^2^_adj_
	
Benzene	10	140 ± 6.6	118 ± 23	3.7 × 10^1^	0.94	260 ± 5.7	263 ± 19	1.1 × 10^−4^	0.96
	8	166 ± 1.9	147 ± 6.0	6.7 × 10^−3^	0.99	160 ± 1.1	148 ± 8.9	5.1 × 10^−3^	0.99
	5	175 ± 5.1	171 ± 9.3	7.5 × 10^−4^	0.96	160 ± 4.8	148 ± 9.2	1.5 × 10^−3^	1
	
Toluene	10	127 ± 1.4	NA	7.7 × 10^−6^	0.74	126 ± 3.7	105 ± 8.7	8.9 × 10^−3^	0.97
	8	128 ± 1.1	91 ± 23	6.8 × 10^2^	0.93	138 ± 1.5	117 ± 23	4.4 × 10^4^	0.87
	5	57 ± 2.9	NA	2.1 × 10^−5^	0.66	72 ± 2.2	53 ± 10	9.5 × 10^−4^	0.87
	
Xylene	10	125 ± 2.1	93 ± 20	9.0 × 10^4^	0.92	132 ± 14	110 ± 21	8.7 × 10^3^	0.94
	8	NA	NA	3.6 × 10^−4^	0.38	101 ± 1.1	109 ± 16	3.3 × 10^−4^	0.89
	5	102 ± 2.6	120 ± 8.9	1.0 × 10^−4^	0.97	100 ± 1.6	92 ± 12	2.2 × 10^−4^	0.96

**Table 3.** Parameters of Freundlich adsorption isotherms.

Chemical	EVB			EVB-SMCH		
	K_f_	n	r^2^	K_f_	n	r^2^
Benzene	1.1 ± 0.28	0.56 ± 0.18	0.89	2.3 ± 1.4	1.4 ± 0.28	0.85
Toluene	6.9 ± 1.0	0.73 ± 0.08	0.92	5.3 ± 0.45	0.82 ± 0.06	0.94
Xylene	5.4 ± 0.47	1.2 ± 0.30	0.85	3.3 ± 0.40	1.9 ± 0.55	0.85

## Data Availability

Data is available upon request.
