# Supplementary material for: Green-Engineered Barrier Creams with Montmorillonite-Chlorophyll Clays as Adsorbents for Benzene, Toluene, and Xylene
Source: Separations. Author manuscript; Available in PMC 2023 May 26. (PMC10214870; doi:10.3390/separations10040237)
Supplement: Supplementary Material [file NIHMS1898562-supplement-Supplementary_Material.docx]

**Green-engineered Barrier Creams with Montmorillonite-chlorophyll Clays as Adsorbents for Benzene, Toluene, and Xylene**

*Meichen Wang, Timothy D. Phillips^*^*

Department of Veterinary Physiology and Pharmacology, College of Veterinary Medicine and Biomedical Sciences, Texas A&M University, College Station, TX 77843, USA

**^*^**Correspondence: [tphillips@cvm.tamu.edu](mailto:tphillips@cvm.tamu.edu); +1 (979)845-6414

*

**Figure S1.** Effect of EVB formulations on *L. minor* (A) frond number, (B) surface area of surviving plants, (C) chlorophyll content on day 7, and (D) growth rate (bar graph) and inhibition percentage (line) (^*^p≤0.05 compared to media control).

**Figure S2**. Effect of EVB formulations on hydra morphology. The hydra media control showed consistent scores of 10 and no significant difference was observed with the inclusion of EVB formulations at up to 0.5% inclusion in the hydra media. Data represent the mean morphological score at each time point, run in triplicate.
